# Death on the Floor

**Published:** 1871-05

**Authors:** 


### DEATH ON THE FLOOR.

SERIOUS and even fatal illness has sometimes followed getting up from a warm bed and stepping on a plank or stone floor, or upon oil-cloth; to place the bare feet on a carpet and walking about, even for a few minutes, endangers illness, especially in feeble constitutions. Parents are often called up suddenly in the night to attend to the wants of infant children. Inconvenience, discomfort, and troublesome sickness can be avoided by having a pair of cloth slippers at the bedside, large enough to admit of a thick layer of wool to be attached to the bottom and sides; they can be instantly put on, even in the dark.

